# Risk perception and generative AI discontinuance intention: mediating effect of cognitive fatigue and moderating effect of AI dependence

**DOI:** 10.3389/fpsyg.2026.1879080

**Published:** 2026-08-10

**Authors:** Wenwen Zhao, Jiawen Liu

**Affiliations:** School of Literature and Journalism, Guangdong Ocean University, Zhanjiang, China

**Keywords:** AI anxiety, AI dependence, AI discontinuation intention, cognitive fatigue, generative artificial intelligence, risk perception

## Abstract

With the rapid popularization of generative artificial intelligence (AI), the risks of misinformation dissemination and user privacy leakage have become key factors influencing users' decisions on the continuous use of AI. Most existing studies focus on the driving mechanisms of AI adoption, while there is insufficient exploration of the reasons for negative usage outcomes, especially the reasons for users to discontinue using the technology. Based on the cognitive-affective-behavioral (C-A-B) framework, this study analyzes the data from 426 valid questionnaires to explore how risk perception affects users' intention to discontinue using AI, identify the mediating role of cognitive fatigue, and the moderating role of AI dependence—which refers to users' deep psychological and behavioral attachment to AI tools and differs from casual habitual use and general technology reliance. The study finds that both the misinformation risk perception and privacy risk perception significantly increase AI anxiety, and this effect is further strengthened by increased misinformation fatigue and privacy fatigue. Meanwhile, AI anxiety significantly promotes users' intention to discontinue using AI, and AI dependence has a negative moderating effect on this relationship. Therefore, cognitive fatigue is an important mediating mechanism connecting risk perception and affective response, and affective factors are key antecedent variables driving users' technology discontinuation intention.

## 1 Introduction

The period from 2022 to 2025 marked an explosive growth phase for artificial intelligence. AIGC technologies, anchored in large-scale models, underwent rapid iteration and achieved widespread adoption. Intelligent tools like ChatGPT have become deeply embedded in users' search queries, content writing, knowledge acquisition, creative production, and corporate operational workflows, emerging as critical digital infrastructure that drives both personal productivity gains and industrial transformation. According to the Europol Innovation Lab (2022), AI-generated content could account for 90% of all internet content by 2026.

The massive proliferation and hyper-realistic quality of AI-generated content (AIGC) are precipitating severe societal challenges, including the widespread dissemination of misinformation, cyberspace disorder, and the erosion of social trust (Yao and Shen, 2020). A scoping review analyzing 24 relevant empirical studies found that AIGC is capable of producing highly deceptive false content, thereby engendering user trust confusion (Park and Nan, 2026). Relative to conventional digital products, AI tools present substantially more pronounced privacy risks, wherein characteristics such as uncontrollability, non-accountability, and technical opacity intensify user insecurity (Mao et al., 2025). Within AIGC-driven information environments, users' continuous engagement in information authenticity verification precipitates cognitive fatigue (Wang and Lu, 2026), which subsequently triggers negative affective responses, including anxiety (Pang et al., 2026), and ultimately attenuates willingness to adopt these technologies (Tencent Research Institute, 2025). Recent incidents involving AI-enabled face-swapping violations and the automated production of toxic content have further intensified public apprehension, underscoring the exigent necessity for robust AI governance frameworks.

While existing scholarship has extensively examined technology adoption and sustained usage, scholarly attention to technology disengagement remains comparatively limited. User discontinuation behaviors typically emerge from risk perception, technological pressure, and the accumulation of negative emotions (Maier et al., 2015), reflecting a complex dynamic process shaped by both risk-related and affective factors. Within AI contexts, where misinformation risks coexist with privacy uncertainties, users experience heightened psychological stress and encounter increased complexity in risk assessment, rendering the mechanisms underlying disengagement intentions particularly critical. However, current research on “AI discontinuation intention” remains notably deficient, with negative usage behaviors and their underlying psychological mechanisms yet to receive adequate scholarly attention.

More critically, the transformation from risk perception to behavioral decision-making is not direct. In the process through which misinformation and privacy risks influence user behavior, cognitive burdens generated during information evaluation and risk assessment—such as recognition fatigue—may further trigger negative affective responses (e.g., AI anxiety), which subsequently shape technology adoption decisions through cognitive-affective mechanisms. When users consistently encounter high-risk and highly uncertain digital content, they may experience affective exhaustion and cynicism fatigue, ultimately influencing their behavioral choices (Choi et al., 2018). In AI environments, identifying misinformation and assessing privacy risks require sustained cognitive resource investment, leading to cognitive fatigue and, consequently, intentions to disengage. However, this “cognitive-affective-behavioral” mechanism has yet to be empirically validated in a systematic manner.

Furthermore, heterogeneity in user behavioral responses cannot be overlooked. High-dependency users typically exhibit stronger tendencies toward continued use, demonstrating lower susceptibility to converting negative emotions into discontinuation intentions relative to low-dependency users. This suggests that AI dependency may function as a critical boundary condition in understanding the relationship between anxiety and discontinuation intentions.

In summary, against the backdrop of rapid AI tool proliferation, how do user-perceived risks of misinformation and privacy concerns jointly influence usage decisions? Does cognitive fatigue serve as a mediating mechanism in this process? Can AI dependency either attenuate or amplify the adverse effects of anxiety-related emotions? By addressing these questions, this study aims to systematically elucidate the mechanisms and boundary conditions underlying AIGC users' discontinuation intentions, thereby enriching existing scholarship in the field.

## 2 Literature review

### 2.1 Cognitive-affective-behavioral model

The Cognitive-Affective-Behavioral (C-A-B) model provides a robust theoretical lens for understanding users' technology adoption decisions in risk environments. Its central premise is that external stimuli first trigger cognitive evaluations (such as risk assessments), which then elicit affective responses and ultimately drive the formation and modification of behavioral intentions (Lazarus, 1982). External information can trigger affective reactions, which may contribute to the development of attitudes and behaviors (Zajonc, 1980). Bagozzi (1992) further refined this model, proposing that individuals typically form a self-regulation sequence involving “cognitive evaluation-affective response-behavioral adjustment” when confronting complex events. In this sequence, emotions play a pivotal role after cognitive processing by influencing behavioral intentions and specific responses. Owing to its explanatory power, the C-A-B framework has been extensively applied to elucidate user behavior mechanisms under various digital risks.

Grounded in the cognitive-affective-behavioral (C-A-B) framework, existing scholarship has elucidated how users formulate continuation or discontinuation decisions under conditions of digital risk, uncertainty, and technological pressure. When confronted with complex technologies, users initially construct cognitive assessments of technological threats, risks, and perceived control. This cognitive appraisal process subsequently triggers affective responses, including anxiety, tension, and frustration, which ultimately manifest as behavioral outcomes such as avoidance, abandonment, or reduced usage intensity (Beaudry and Pinsonneault, 2010). Specifically, individuals undergo two sequential stages when encountering potential threats: “primary appraisal” (assessing whether a threat exists) and “secondary appraisal” (determining whether the threat can be effectively managed) (Lazarus and Folkman, 1984). This risk judgment process induces affective experiences encompassing fear, concern, and fatigue (Smith and Lazarus, 1990). In digital risk scenarios, emotions frequently serve as pivotal drivers of behavioral adaptation, particularly when users confront privacy threats, technological uncertainties, or potential erroneous outputs, wherein emotions assume a significant mediating function (Perrewé et al., 2012). Collectively, these studies demonstrate that risk perception is correlated with affective states in digital technology usage contexts, while emotional experiences directly determine whether users adopt avoidance or withdrawal behaviors.

With the rapid advancement of generative AI technologies, the C-A-B model has been increasingly integrated into analyses of AI usage behavior, thereby extending its applicability to emerging technological contexts. When confronted with the black-box nature of AI systems and their uncontrollable, opaque generation mechanisms, users experience substantial cognitive uncertainty. This uncertainty subsequently triggers affective responses, including anxiety, apprehension, and psychological disequilibrium, ultimately leading to reduced usage frequency or discontinuation intentions (Zhou and Zhang, 2025). The difficulty inherent in discerning AI-generated content elevates cognitive load, inducing fatigue and affective exhaustion (Kim et al., 2025), which may in turn prompt users to adopt avoidance or withdrawal behaviors. Similarly, awareness of privacy risks engenders anxiety, irritability, and fatigue (Choi et al., 2018), which may elevate discontinuation intentions among digital product users.

This study proposes a refined cognitive-affective-behavioral (C-A-B) explanatory framework. Within generative AI contexts, user judgments regarding misinformation and privacy risks constitute cognitive appraisals. The persistent allocation of cognitive resources to mitigate these risks may precipitate cognitive fatigue—a hybrid state encompassing both cognitive depletion and affective strain. Accumulated fatigue may subsequently trigger AI-related anxiety, potentially resulting in reduced usage frequency or discontinuation intentions. Accordingly, this study conceptualizes misinformation and privacy risks as cognitive antecedents, AI cognitive fatigue (comprising misinformation fatigue and privacy fatigue) as a mediating mechanism, AI-induced anxiety as an affective mediator, and AI discontinuation intention as the behavioral outcome.

### 2.2 AI risk perception, AI cognitive fatigue, and AI anxiety

The essence of AI risk perception resides in users' subjective judgments regarding potential harm, losses, and uncertainties. Extant research indicates that when evaluating novel technologies, users frequently perceive multiple risks simultaneously, encompassing functional, ethical, privacy, and information authenticity dimensions. The rapid proliferation of AIGC technologies has further heightened the structural complexity of these risk configurations (Pang et al., 2026). This study focuses on two core dimensions: misinformation risk perception and privacy risk perception.

Misinformation risk perception refers to an individual's subjective concern regarding, and perceived likelihood of encountering, false, misleading, or unverified information, along with the potential adverse consequences of such exposure. However, a clear conceptualization of AI-related misinformation risk perception remains absent from the extant literature. Accordingly, by extending the construct of misinformation risk perception to AI contexts, this study proposes the following definition: AI misinformation risk perception denotes users' subjective concerns regarding potential factual errors, misleading content, and authenticity uncertainties inherent in AI-generated outputs. With the widespread proliferation of large language models (LLMs), the scale, speed, and linguistic fluency of AI-generated content have increased substantially (Sun et al., 2024), rendering authenticity assessment increasingly arduous for users. The phenomenon whereby misinformation manifests within AI systems is termed AI hallucination (Sun et al., 2024). Drawing on Pennycook and Rand's (2021) work, the study argues that content produced by generative AI frequently exhibits high reading fluency and logical coherence, which may obscure factual inaccuracies and thereby elevates users' risk perception levels. Experimental evidence reveals that when encountering texts generated by large models, users' ability to detect their artificial origin is impaired, which may elevate their perceived concerns over risks such as deception and fraud (Jakesch et al., 2023).

Privacy risk perception refers to users' subjective awareness of potential losses resulting from the unauthorized collection, use, or disclosure of their personal information in the context of information service utilization (Featherman and Pavlou, 2003). However, a clear conceptualization of AI privacy risk perception remains absent from the extant literature. Building upon the construct of privacy risk perception, AI privacy risk perception is defined herein as users' subjective concerns regarding potential losses arising from the unauthorized collection, improper use, or inadvertent disclosure of personal data during the utilization of generative AI tools. The capacity of artificial intelligence technologies to harness vast quantities of rich data has precipitated substantial privacy and anonymity concerns (Akakpo et al., 2025).

The risks associated with AI—including erroneous outputs, privacy concerns, and opaque automated decision-making—can provoke substantial affective distress, fear, anxiety, and perceived threats (Kim et al., 2025; Wang and Wang, 2022). Consequently, individuals increasingly experience a complex psychological state characterized by the coexistence of dependence and resistance, namely AI anxiety (Johnson and Verdicchio, 2017; Pang et al., 2026). Technological anxiety refers to the fear or discomfort experienced by individuals when using technology (Nurtanto et al., 2025). However, unlike other forms of technology-related anxiety, AI anxiety refers to the fear or anxiety caused by the potential loss of control over AI, and AI anxiety primarily stems from misconceptions about technological advancement, confusion regarding AI autonomy, and blindness to socio-technical systems (Johnson and Verdicchio, 2017). Besides, AI anxiety exhibits distinct contemporary features: more complex etiological triggers, heightened intensity, and frequent onset attributable to algorithmic black-box mechanisms, ambiguous content authenticity, and insufficient data control (Pang et al., 2026).

Anxiety exhibits an “affective amplification effect”—wherein stronger perceived risk more readily triggers anxious responses (Pang et al., 2026). Concerns regarding authenticity in AIGC, the opacity of algorithmic black boxes, and uncontrollable privacy risks collectively shape users' affective responses, generating worry, unease, and even fear during interactions (Chen and Li, 2025). Content produced by generative AI frequently exhibits high reading fluency and logical coherence, which may obscure factual inaccuracies and thereby elevates users' risk perception levels (Pennycook and Rand, 2021). The system's lack of explainability directly activates users' affective threat responses, engendering persistent uncertainty and psychological pressure during AI interactions (Magni et al., 2024). Similarly, concerns regarding data misuse for unspecified purposes intensify privacy anxiety, leading to elevated psychological stress levels (Acquisti et al., 2015). Salam et al. (2025) incorporated privacy concerns into the ethical governance framework of intelligent applications. They pointed out that users' perceived privacy risk can significantly change emotional perception and further strengthen users' resistance and avoidance behavior toward AI-enabled services. On this basis, this study further clarifies that when users perceive privacy and information risks, they are prone to ethical discomfort and psychological anxiety, which eventually lead to negative outcomes. Based on these findings, this study proposes the following hypotheses:

H1: AI misinformation risk perception is positively associated with AI anxiety.

H2: AI privacy risk perception is positively associated with AI anxiety.

Cognitive fatigue denotes a psychobiological state that emerges from sustained or high-intensity cognitive task engagement, characterized by a subjectively experienced sense of exhaustion and diminished mental energy (Ezzedini, 2026). Accordingly, AI cognitive fatigue refers to a state of psychological exhaustion and cognitive resource depletion experienced by users within generative AI environments. In such contexts, users must continuously exert cognitive effort to assess information authenticity. Although the high fluidity and readability of AI-generated content may appear to reduce processing demands, they actually intensify the difficulty of thorough verification (Park and Nan, 2026). This compels users to expend greater cognitive effort, thereby exacerbating fatigue. When users are unable to determine the authenticity of AI-generated content, accumulated judgment costs result in sustained psychological fatigue (Kim et al., 2025). Moreover, the ambiguous nature of AI-generated content substantially elevates cognitive barriers during verification tasks (Chen and Li, 2025).

In the context of an increasingly challenging privacy environment and complex personal data protection measures, frequent data breaches have exacerbated users‘ doubts about their ability to safeguard their privacy. When individuals perceive themselves as incapable of effectively managing intricate privacy protection tasks, they are likely to experience significant psychological stress and a sense of helplessness, ultimately leading to privacy fatigue (Choi et al., 2018). Privacy fatigue reflects a state of emotional exhaustion and cynicism toward privacy management, which substantially influences both privacy protection behaviors and disclosure intentions (Choi et al., 2018; Chung and Kwon, 2025). Empirical evidence supports this view. For instance, Rajaobelina et al. (2021) observed that users felt shocked and helpless upon discovering how insurance companies' chatbots could utilize limited personal information to construct highly detailed profiles, resulting in pronounced fatigue. Similarly, Hinds et al. (2020) reported that despite expressing privacy concerns, users often considered protective actions futile, further reinforcing the onset of privacy fatigue. In scenarios marked by dual uncertainties—pertaining to both information authenticity and privacy security—users become particularly vulnerable to sustained cognitive strain and psychological exhaustion. Based on this reasoning, the study proposes the following hypotheses:

H3: AI misinformation risk perception is positively associated with users' misinformation fatigue.

H4: AI privacy risk perception is positively associated with users' privacy fatigue.

AI-induced fatigue not only depletes cognitive resources but also elicits negative affective responses. The capacity of artificial intelligence to effortlessly generate persuasive misinformation (Zhou et al., 2023) has transformed the internet into a fertile ground for fake news and disinformation (Park, 2024). Detecting AI-generated misinformation entails considerable cognitive effort, and this sustained vigilance can lead to significant psychological fatigue (Kim et al., 2025). When cognitive resources are depleted and individuals enter a state of fatigue, their attentional bias toward environmental threats intensifies, impairing affective regulation and elevating anxiety levels (Eysenck et al., 2007). Similarly, the complexity and uncertainty inherent in privacy risks amplify individuals' information processing burdens (Acquisti et al., 2015). The intricacy of privacy management may induce privacy fatigue (Kwon and Johnson, 2015), which in turn exacerbates fear, negative emotions, and perceptions of uncertainty (van der Schyff et al., 2023). Supporting this pathway, an empirical study conducted in the context of climate change risk found that privacy concerns related to AI technology significantly heighten public AI anxiety (Hong, 2025). Overall, stress arising from technological overload, complexity, and perceived insecurity constitutes a critical antecedent of AI anxiety (Pang et al., 2026). Based on this integrated reasoning, the study proposes the following hypotheses:

H5: AI misinformation fatigue is positively associated with AI anxiety.

H6: AI privacy fatigue is positively associated with AI anxiety.

In summary, the perception of misinformation risks contributes to AI-induced cognitive fatigue, which subsequently fosters AI anxiety. This observation aligns with Issa et al. (2024), who found that AI information involving privacy risks stimulates users' threat perception, thereby triggering anxiety in AI usage. Ayyagari et al. (2011) demonstrated that technological characteristics can trigger technostress such as perceived privacy infringement, thereby exerting adverse psychological outcomes on users. Zhou and Zhang (2025) further indicated that privacy concerns and AI-generated information hallucinations induce cognitive dissonance in users, leading to their intermittent discontinuation of AI use.

Cognitive load theory posits that human cognitive capacity is finite and subject to influence from both internal and external factors (Sweller, 1988). When cognitive demands exceed an individual's processing capacity, cognitive overload and exhaustion ensue, which subsequently precipitate negative emotional responses. When users perceive that AI-generated outputs may contain misinformation or privacy violations, they must allocate substantial cognitive resources to verification and evaluation. The resultant cognitive fatigue then triggers AI anxiety. Collectively, these findings suggest that the cognitive burden experienced by AI users serves as an intermediary mechanism linking stressors to affective and behavioral outcomes.

Building on this reasoning and the theoretical framework established earlier, the study formally proposes that AI misinformation fatigue serves as a mediating mechanism between misinformation perceptions and AI anxiety. Correspondingly, AI privacy fatigue is posited to mediate the relationship between privacy risk perception and AI anxiety. These two fatigue constructs are specified as parallel mediators. Thus, the following hypotheses are advanced:

H7: AI misinformation fatigue mediates the relationship between misinformation perceptions and AI anxiety.

H8: AI privacy fatigue mediates the relationship between privacy risk perception and AI anxiety.

### 2.3 AI anxiety, AI discontinuance intention, and AI dependence

Technical discontinuance intention typically refers to users' behavioral tendency to reduce or cease system usage during information system utilization, triggered by technical stress, cognitive load, or negative experiences (Zhou and Zhang, 2025). This concept originates from information system retention theory, with theoretical foundations tracing back to Bhattacherjee (2001) expectation-confirmation model, often regarded as the inverse behavioral outcome of retention intention in research. In this study, AI discontinuance intention is interpreted as users' withdrawal tendencies formed after experiencing risk perception, cognitive load, and affective stress. Compared to technology adoption and retention, discontinuance intention has received less attention in early research. However, as digital issues such as technical pressure, information overload, and privacy breaches have become more prominent, discontinuance behavior has gradually emerged as a research focus. When users encounter highly uncertain and risky technologies, negative emotions rapidly accumulate, directly driving behavioral withdrawal to avoid prolonged exposure to threatening scenarios (Beaudry and Pinsonneault, 2010; Perrewé et al., 2012).

Anxiety serves as a key mechanism driving discontinuance behaviors (Wang et al., 2025). It impairs goal-directed attention control, increases reliance on stimulus-driven attention, and reduces the effective utilization of cognitive resources, thereby compromising processing efficiency in complex cognitive tasks (Eysenck et al., 2007). High levels of AI anxiety significantly diminish users' trust, resulting in pronounced avoidance tendencies (Tao et al., 2025). In generative AI environments, anxiety rapidly accumulates when users encounter risks such as uncertain authenticity, unmanageable data, or algorithmic errors, manifesting as reduced usage frequency, diminished dependency, or even intentions to disengage (Liu et al., 2025). Based on these theoretical frameworks and empirical evidence, this study proposes:

H9: AI anxiety is positively associated with users' AI discontinuance intention.

Artificial intelligence (AI) dependence refers to a behavioral reliance in which individuals excessively use AI technology, potentially developing into uncontrollable behavior with addictive characteristics (Frenkenberg and Hochman, 2025). Its formation is not instantaneous but a gradual process rooted in behavioral inertia: repeated engagement with a specific technology can lead to path dependence and lock-in effects (Arthur, 1989), making the technology increasingly perceived as indispensable and ultimately consolidating initial habitual use into dependence (Frenkenberg and Hochman, 2025).

This state of dependence moderates the impact of AI anxiety on discontinuance intention through a dual mechanism. On the one hand, dependence weakens users' alertness to risks (Eysenck et al., 2007) and leads to “cognitive-behavioral decoupling” (Limayem et al., 2007), making it difficult for anxiety to translate into actual discontinuance decisions. On the other hand, AI dependence is related to positive usage motivations such as perceived utility, self-efficacy, and intrinsic interest in AI technology. These factors jointly encourage users to use AI systems more deeply and persistently, which collectively promote deeper and more sustained user engagement with AI systems, thereby attenuating discontinuance intention (Frenkenberg and Hochman, 2025). Therefore, the higher the degree of AI dependence, the weaker the positive effect of anxiety on discontinuance intention. Accordingly, this study proposes the following hypothesis:

H10: AI dependence negatively moderates the effect of AI anxiety on AI discontinuance intention. Specifically, the higher the user's AI dependence level, the weaker the positive influence of AI anxiety on discontinuation intention.

**Figure 1:**
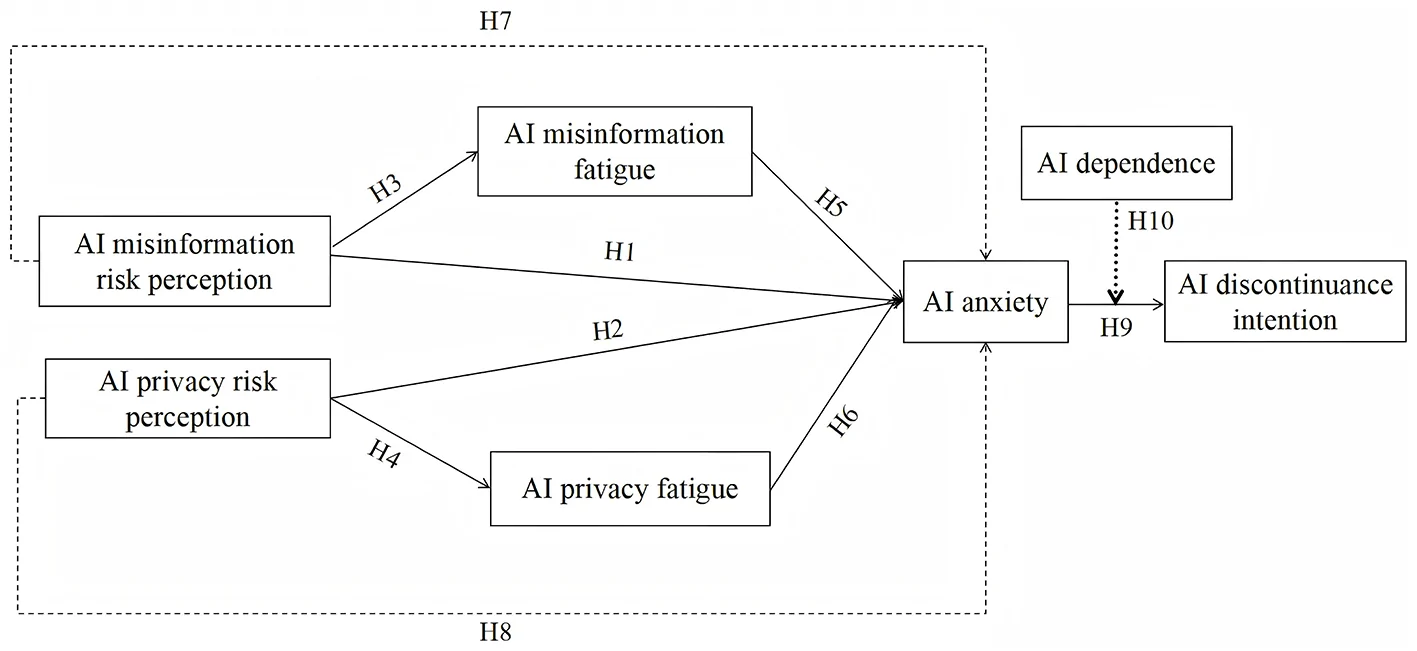
A proposed conceptual framework.

The research model of this paper is shown in Figure 1.

## 3 Research methods

### 3.1 Respondents

This study utilized an online questionnaire for data collection. The survey was designed and administered via the Wenjuanxing platform and disseminated through social media channels. A total of 500 responses were initially gathered. Following a data-cleaning procedure that excluded invalid submissions based on excessively short completion times, repetitive response patterns (over 80% identical answers), obvious logical inconsistencies, or outlier values, 426 valid questionnaires were retained for analysis. As shown in Table 1, the sample's demographic profile indicates that 65.3% (*n* = 278) of respondents were female, and 34.7% (*n* = 148) were male. The sample was predominantly young, with 80% aged between 18 and 25 years, and 14.8% aged 26–35, reflecting the prevalence of young adults among current generative AI users. In terms of educational attainment, most participants held a bachelor's degree (72.1%), followed by those with postgraduate qualifications (master's or doctoral degrees) at 16.2%.

**Table 1:** Demographic characteristics of the survey samples (*N* = 426).

Variables	Items	*N*	Percentage (%)
Gender	Man	148	34.7
	Woman	278	65.3
Age	25 years old and below	348	81.7
	26–35 years old	63	14.8
	36–45 years old	12	2.8
	Over 45 years of age	3	0.7
Educational level	High school and below	50	11.7
	Undergraduate students	307	72.1
	Graduate students (Master's and Doctoral level)	69	16.2
AI operating frequency	Several times a day	82	19.2
	Often (almost every day)	132	31.0
	Sometimes (3–4 times per week)	143	33.6
	Occasionally (1–2 times per week)	54	12.7
	Rarely (less than once per week)	15	3.5
AI functional usage patterns	Writing, drafting, and polishing text	310	72.8
	Information retrieval/finding materials	285	66.9
	Text summarization/summarizing key points	246	57.7
	Chatting, companionship, and entertainment	208	48.8
	Translation/language learning	184	43.2
	Creative content generation	121	28.4
	Data analysis/formula suggestion	89	20.9
	Code generation/debugging	87	20.4
	Information verification	60	14.1
	Others	63	15.4

Regarding the frequency of AI usage, the largest proportion of respondents reported using AI almost daily. This indicates a high level of AI exposure and practical experience within the sample, thereby enhancing the study's capacity to capture authentic risk perception and psychological responses in real-world contexts. In terms of functional applications, respondents' use of generative AI demonstrated considerable diversity. The most prevalent application was writing, drafting, and text polishing (72.8%), followed by information retrieval and data searching (66.9%), text summarization (57.7%), as well as learning assistance and translation. Additionally, a portion of respondents reported employing AI for more complex tasks such as code generation, data analysis, creative content generation, and automated office work.

### 3.2 Measurement

This study investigates user behavior in generative artificial intelligence (AI) contexts, with a focus on the mechanisms through which risk perception—specifically regarding misinformation and privacy—influence discontinuation intention. It further examines the mediating roles of AI anxiety and AI cognitive fatigue, as well as the moderating effect of AI dependence. The survey instrument operationalized seven core constructs, along with demographic variables: (1) AI misinformation risk perception, (2) AI privacy risk perception, (3) AI anxiety, (4) AI misinformation fatigue, (5) AI privacy fatigue, (6) AI discontinuation intention, (7) AI dependence, and (8) demographic information. Core constructs were adapted and employed a seven-point Likert scale (1 = strongly disagree, 7 = strongly agree).

All constructs in this study reflect subjective psychological states, which are most accurately measured via respondents' self-reports. Indeed, self-report methodology is well established as the gold standard for assessing subjective psychological constructs (Corneille and Gawronski, 2024). Given the cross-sectional design of this study, which focuses on capturing behavioral intentions rather than realized behaviors, objective behavioral indicators (e.g., actual discontinuance of AI tools) were not feasible for this analysis. To further ensure the construct validity of our measurements, all constructs were operationalized using well-established scales adapted from prior literature.

The construct of AI misinformation risk perception captures users' subjective assessment of potential harms associated with AI-generated content, such as factual inaccuracies, misleading claims, and challenges in authenticity verification. Measurement items were adapted from Roozenbeek et al. (2022). AI privacy risk perception refers to users‘ subjective evaluation of potential privacy threats, including unauthorized collection, storage, misuse, or leakage of personal data. Items were drawn from Featherman and Pavlou (2003). AI anxiety refers to the negative emotional responses (e.g., tension, worry, and perceived threat) arising from the perceived uncontrollability of AI technology, measured using items adapted from Wang and Wang (2022).

AI cognitive fatigue, comprising AI misinformation fatigue and AI privacy fatigue, describes a state of cognitive overload, psychological exhaustion, and diminished willingness to engage in judgment tasks arising from processes of information verification and risk assessment. Items were adapted from Choi et al. (2018). AI discontinuance intention gauges users' inclination to reduce or terminate their use of AI tools, with items sourced from Maier et al. (2015) and Bhattacherjee (2001). AI dependency assesses the degree of users' established usage inertia and reliance on AI, measured using items adapted from Huang et al. (2024). All measurement items are presented in Table 2.

**Table 2:** Measurement items.

Variables	items
AI misinformation risk perception	I'm concerned about the lack of transparency in the sources of AI-generated content, making it hard to assess its reliability.
	I believe AI-generated responses may contain outdated or incorrect information.
	I am concerned that AI-generated information lacks reliable evidence or citation support.
	I believe that even if AI-generated content is grammatically correct, it may still contain hidden misinformation.
	I'm concerned that AI-generated content lacks clearly defined responsible parties, making it difficult to trace its origins.
	I would question whether the real author of AI-generated content or the underlying algorithm is trustworthy.
	I believe AI tools may generate information that appears credible but is actually unreliable.
	I often worry that AI-generated content requires further verification to confirm its authenticity.
	I believe the format and presentation style of AI-generated content may mislead people in assessing its authenticity.
AI privacy risk perception	Using AI tools may result in me losing control over my personal information.
	I'm concerned that my personal information might be exploited by AI systems.
	AI tools may disclose my personal information to third parties without my consent.
	Overall, I'm concerned that using AI tools might compromise my privacy.
AI misinformation fatigue	Repeated verification of the authenticity of AI-generated information has left me affectively exhausted.
	Dealing with the authenticity issues of AI-generated content has left me utterly exhausted.
	I'm tired of constantly evaluating whether AI-generated information is trustworthy.
	It's exhausting to constantly monitor misleading or misinformation generated by AI tools.
	I've lost interest in assessing the reliability of AI-generated content.
	I have become less proactive in identifying misinformation generated by AI.
	I doubt whether my efforts to verify the accuracy of AI-generated information have any real impact.
	Sometimes, I feel that worrying about the accuracy of AI-generated information is pointless.
AI Privacy Fatigue	I'm affectively drained from constantly monitoring how AI tools handle my personal data.
	Managing privacy settings and authorization requests for AI applications has left me utterly exhausted.
	I am tired of constantly evaluating whether AI systems excessively collect or share personal information.
	The constant need to monitor data security on the AI platforms I use has left me exhausted.
	I've lost interest in how surveillance AI systems handle my privacy.
	When using AI technology, I have become less proactive about protecting personal data.
	I'm skeptical whether adjusting privacy settings in AI tools will actually make any difference.
	Sometimes I feel that focusing on data privacy in AI systems is utterly pointless.
AI anxiety	I am concerned that AI technologies/products may be misused.
	I am concerned about various potential issues related to AI technology/products.
	I'm concerned that AI technologies/products might lose control and malfunction.
	I am concerned that AI technologies/products may lead to the autonomy of robots.
	I find anthropomorphic AI technologies/products truly unsettling.
	I find anthropomorphic AI technologies/products truly intimidating.
	I can't quite explain why, but anthropomorphic AI technologies/products make me uneasy.
	I'm concerned that AI technologies/products might lead to dependency.
AI discontinuance intention	I plan to stop using AI tools in the near future.
	I think I might stop using AI tools soon.
	I might stop using AI tools altogether.
	I plan to gradually reduce my reliance on AI tools.
	I might switch to non-AI methods to replace AI tools.
AI dependence	I spend way too much time using AI.
	The use of AI has damaged my relationships with others.
	When I cannot use AI, I feel anxious and uneasy.
	I tried to reduce my AI usage time, but it didn't work out.

## 4 Research findings

### 4.1 Measurement model analysis

This study assessed the internal consistency of the scales using Cronbach's α coefficient and evaluated the measurement model through confirmatory factor analysis (CFA) to examine convergent and discriminant validity. The results (see Table 3) indicated that all variables had Cronbach's α values above 0.8, suggesting high reliability. The average variance extracted (AVE) for each construct exceeded 0.5, and composite reliability (CR) values were above the recommended threshold of 0.7, supporting satisfactory convergent validity. Discriminant validity was tested using the heterotrait-monotrait ratio (HTMT). HTMT values represent the true correlation between constructs, with values below 0.90 indicating adequate discriminant validity. In the study, all HTMT values in this study were well below this threshold, confirming discriminant validity. Therefore, the measurement model demonstrates good psychometric properties, and the data are suitable for further analysis.

**Table 3:** Reliability and validity of the measurement.

Variables	1	2	3	4	5	6	CR	Cronbach'α	AVE	Loadings
AIMRP							0.942	0.930	0.643	0.745–0.841
AIPRP	0.516						0.916	0.877	0.731	0.817–0.879
AIMF	0.482	0.491					0.926	0.910	0.610	0.680–0.843
AIPF	0.411	0.462	0.717				0.918	0.899	0.585	0.703–0.812
AIA	0.516	0.636	0.597	0.582			0.916	0.895	0.577	0.700–0.861
AIDI	0.103	0.255	0.287	0.346	0.306		0.956	0.943	0.814	0.875–0.919
AID	0.293	0.318	0.355	0.364	0.348	0.341	0.865	0.843	0.566	0.700–0.861

AIMRP, AI misinformation risk perception; AIPRP, AI privacy risk perception; AIMF, AI misinformation fatigue; AIPF, AI privacy fatigue; AIA, AI anxiety; AIDI, AI discontinuance intention; AID, AI dependence.

Common method variance was examined using Harman's single-factor test. The unrotated principal components analysis revealed that the largest factor explained 31.23% of the variance, which is below the 40% threshold suggested by Podsakoff et al. (2003). Besides, following Kock (2015) methodological guideline, the full collinearity test was employed to assess common method variance (CMV). A VIF threshold of 3.3 serves as the widely accepted critical cutoff: any latent variable exceeding this value indicates potential CMV bias. In this study, all latent constructs yielded VIF values well below the 3.3 benchmark, ranging from 1.000 to 1.963. Thus, common method bias does not appear to be a significant concern in this study.

### 4.2 Structure model evaluation

Statistical significance and practical magnitude of the hypothesized paths were examined to reveal the mechanisms underlying the observed phenomenon. Model evaluation was conducted based on five primary criteria: (a) significance of path coefficients, (b) the coefficient of determination (*R*^2^), reflecting the model's explanatory power, (c) effect size (*f* ^2^) quantifying the relative strength of relationships between constructs, (d) predictive relevance (*Q*^2^), assessed via blindfolding, and (e) model fit using SRMR.

According to Chin (1998), *R*^2^ values of 0.190, 0.333, and 0.670 indicate weak, moderate, and substantial explanatory power, respectively. As shown in Table 4, the *R*^2^ values for AI misinformation fatigue and AI discontinuance intention were 0.220 and 0.223, respectively, indicating a moderate explanatory power. However, the *R*^2^ for AI anxiety was 0.471, indicating that risk perception and cognitive fatigue contribute a considerable proportion of the variance in AI anxiety.

**Table 4:** Evaluation of Measurement based on *R*^2^, *f*^2^, and *Q*^2^.

Variables	*R* ^2^	*f* ^2^						*Q* ^2^
		AIMRP	AIPRP	AIMF	AIPF	AIA	AIDI	
AIMRP				0.282		0.031		
AIPRP					0.214	0.133		
AIMF	0.220					0.037		0.125
AIPF	0.176					0.048		0.100
AIA	0.471						0.046	0.267
AIDI	0.223							0.175
AID							0.165	

AIMRP, AI misinformation risk perception; AIPRP, AI privacy risk perception; AIMF, AI misinformation fatigue; AIPF, AI privacy fatigue; AIA, AI anxiety; AIDI, AI discontinuance intention; AID, AI dependence.

Effect size computation was executed to ascertain the substantive impact of each exogenous variable on its respective outcome construct, and *f* ^2^ values of 0.020, 0.150, and 0.350 signify small, medium, and large effect sizes, respectively (Cohen, 1988). In the reserach, AI misinformation risk perception on AI misinformation fatigue was moderate (*f*^2^ = 0.282), and the influenec of AI privacy risk perception on AI privacy fatigue was also moderate (*f*^2^ = 0.214), indicating that risk perception exerted medium effects on cognitive fatigue. For AI anxiety, AI misinformation risk perception (*f*^2^ = 0.031), AI misinformation fatigue (*f*^2^ = 0.037), and AI privacy fatigue (*f*^2^ = 0.048) showed small effects, while AI privacy risk perception indicated small to moderate effects (*f*^2^ = 0.133). As for AI discontinuance intention, AI anxiety signifies a small effect (*f*^2^ = 0.046), but AI dependence shows a moderate effect (*f*^2^ = 0.165).

*Q*^2^ values greater than zero confirm predictive relevance (Hair et al., 2017), and SRMR values below 0.08 indicate acceptable model fit (Hu and Bentler, 1999). In the current study, all endogenous constructs exhibited *Q*^2^ values greater than zero, indicating satisfactory predictive relevance. Additionally, the SRMR of 0.075 fell below the recommended threshold of 0.08, suggesting acceptable global model fit. Furthermore, collinearity among predictor constructs was examined using variance inflation factor (VIF) values to ensure the structural model was free from multicollinearity issues.

**Figure 2:**
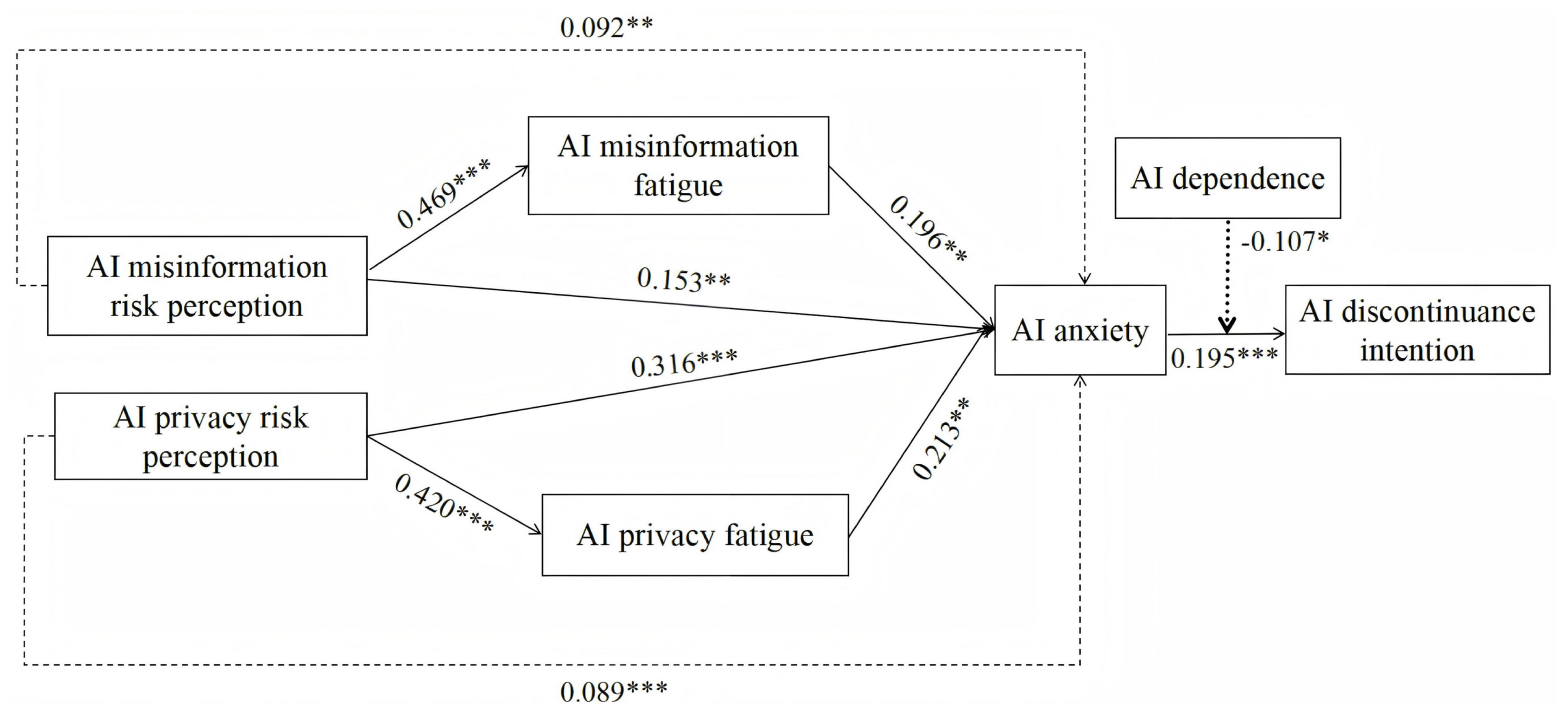
Structural model with standardized estimates. *p < 0.05, **p < 0.01, ***p < 0.001.

This study employed SmartPLS 4.0 software to test the structural model and hypotheses. As shown in Figure 2 and Table 5, the data revealed that AI misinformation risk perception (β = 0.153, *p* = 0.004), AI privacy risk perception (β = 0.316, *p* < 0.001), AI misinformation fatigue (β = 0.196, *p* = 0.003), and AI privacy fatigue (β = 0.213, *p* < 0.001) are positively associated with AI anxiety, supporting H1, H2, H5, and H6. AI misinformation risk perception correlates with AI misinformation fatigue (β = 0.469, *p* < 0.001), suggesting H3. AI privacy risk perception is positively linked to AI privacy fatigue (β = 0.420, *p* < 0.001), suggesting H4. AI anxiety shows a positive correlation with AI discontinuance intention (β = 0.195, *p* < 0.001), supporting H9.

**Table 5:** Results of direct and moderating hypotheses testing.

Hypothesis	β	SE	*p*-value	Result
H1: AI misinformation risk perception → AI anxiety	0.153	0.053	0.004	Supported
H2: AI privacy risk perception → AI anxiety	0.316	0.049	^***^	Supported
H3: AI misinformation risk perception → AI misinformation fatigue	0.469	0.046	^***^	Supported
H4: AI privacy risk perception → AI privacy fatigue	0.420	0.045	^***^	Supported
H5: AI misinformation fatigue → AI anxiety	0.196	0.065	0.003	Supported
H6: AI privacy fatigue → AI anxiety	0.213	0.055	^***^	Supported
H9: AI anxiety → AI discontinuance intention	0.195	0.048	^***^	Supported
H10: AI dependence × AI anxiety → AI discontinuance intention	−0.107	0.044	0.015	Supported

^***^p < 0.001.

**Figure 3:**
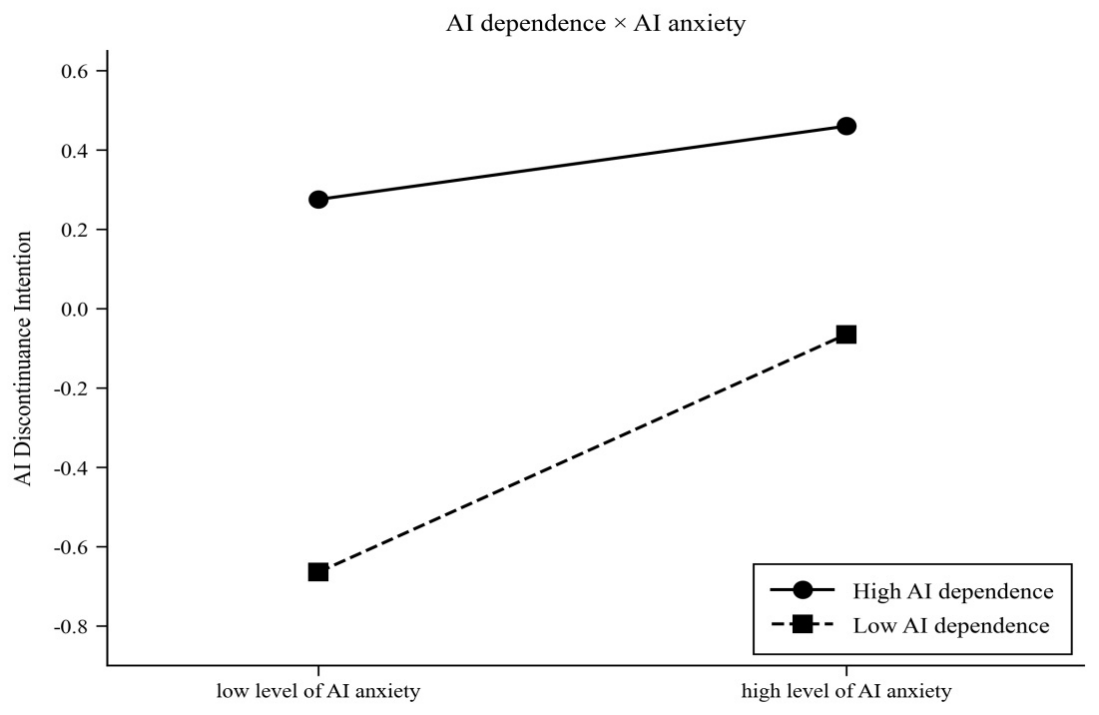
Slope plot of AI dependence moderating AI anxiety on AI discontinuation intention.

The results of the moderation effect path coefficients are presented in Figure 2. AI dependence demonstrates a negative moderating effect on the impact of AI anxiety on AI discontinuance intention (β = −0.107, 95% CI [−0.190, −0.016], *p* = 0.015), confirming H10. Specifically, for users with high AI dependence, the positive influence of AI anxiety on discontinuation intention is significantly attenuated. To visually illustrate the moderation pattern, a simple slope analysis plot (see Figure 3) was constructed. The findings revealed that the positive association between AI anxiety and discontinuance intention was diminished among users with a high level of AI dependence. Consequently, individuals with greater AI dependence were less likely to develop a discontinuance intention in response to AI anxiety. Specifically, when AI dependence was at a low level (−1 SD), AI anxiety exerted a stronger predictive effect on AI discontinuance intention, characterized by a steeper slope. Conversely, when AI dependence was at a high level (+1 SD), AI anxiety had a weaker predictive effect on AI discontinuance intention, accompanied by a more gentle slope.

A total of 5,000 bootstrap samples were used to evaluate the parallel mediation model. As indicated in Table 6, AI misinformation fatigue significantly mediates the relationship between AI misinformation risk perception and AI anxiety (β = 0.092, 95%CI [0.033, 0.160], *p* = 0.004). The total effect of AI misinformation risk perception on AI anxiety (path c) was significant (β = 0.245, *p* < 0.001). As previously demonstrated, the direct effect of AI misinformation risk perception on AI anxiety (path c′) remained significant after controlling for the mediator (β = 0.153, *p* = 0.004). Given that both the indirect effect and the direct effect were statistically significant, partial mediation was confirmed. The variance accounted for (VAF) was 37.6%, indicating that 37.6% of the total effect of AI misinformation risk perception on AI anxiety is transmitted through the indirect pathway of AI misinformation fatigue. Similarly, AI privacy fatigue significantly mediates the relationship between AI privacy risk perception and AI anxiety (β = 0.089, 95%CI [0.042, 0.140], *p* < 0.001). The total effect of AI privacy risk perception on AI anxiety (path c) was significant (β = 0.406, *p* < 0.001). However, after controlling for AI privacy fatigue, the direct effect of AI privacy risk perception on AI anxiety (path c′) remained significant and substantial (β = 0.316, *p* < 0.001). Thus, AI privacy fatigue partially mediated the relationship between AI privacy risk perception and AI anxiety. The VAF was 22.0%, indicating 22% of the total effect was transmitted through the fatigue pathway.

**Table 6:** Results of mediating hypothesis testing.

Hypothesis	Total effect (*c*)	Indirect effect *a* ^*^b	Direct effect *c*^′^	95%CI		*p*	Results
				Lower	Upper		
H7: AIMRP → AIMF → AIA	0.245	0.092	0.153	0.033	0.160	0.004	Supported
H8: AIPRP → AIPF → AIA	0.406	0.089	0.316	0.042	0.140	^***^	Supported

AIMRP, AI misinformation risk perception; AIPRP, AI privacy risk perception; AIMF, AI misinformation fatigue; AIPF, AI privacy fatigue; AIA, AI anxiety; AIDI, AI discontinuance intention; AID, AI dependence. ^***^p < 0.001.

## 5 Discussion

Building upon the “Cognitive-Affective-Behavioral” (C-A-B) framework, this study explores the relationship between AI risk perception, AI cognitive fatigue, AI anxiety, and AI discontinuance intention, considering the moderating role of AI dependence.

H1 and H2 demonstrate that both AI misinformation risk perception and AI privacy risk perception are positively associated with AI anxiety. These findings suggest that risk perception remains a critical antecedent of affective responses in AIGC scenarios. When facing potential threats, individuals activate affective reactions through risk assessment, leading to negative emotions such as anxiety and tension (Smith and Lazarus, 1990; Lazarus, 1999). This finding is consistent with the conclusions of existing studies (Chen and Li, 2025), indicating that algorithmic black boxes, disinformation, privacy breaches, and other related factors significantly exacerbate AI anxiety.

H3 and H4 indicate that both AI misinformation risk perception and AI privacy risk perception are positively linked to cognitive fatigue. When users perceive risks to information authenticity or privacy security, they must allocate additional cognitive resources to judgment and evaluation. From a cognitive load perspective, such processing demands may exceed working memory capacity, resulting in cognitive load (Sweller, 1988). The continuous risk assessment process further exacerbates this state of psychological exhaustion (Choi et al., 2018). AI misinformation fatigue and AI privacy correlate with AI anxiety, thus supporting H5 and H6. The results were consistent with the conclusion of previous studies (Kim et al., 2025; Hong, 2025), AI anxiety was significantly influenced by authenticity and privacy concerns about AI technology.

H7 and H8 suggest that AI cognitive fatigue can explain how AI risk perception influences AI anxiety, supporting the parallel mediation structure. Risk perception not only directly relates to affective responses but also relates to affective states indirectly through higher cognitive load. The conclusions align with the core logic of the C-A-B model, demonstrating how individual cognitive factors affect behavioral outcomes through affective responses (Bagozzi, 1992). However, distinct from traditional direct mechanisms, this study further confirms that risk perception does not directly act on emotions via AI itself, but indirectly influences AI anxiety through cognitive resource depletion mechanisms like cognitive fatigue, revealing a mediating process in cognitive-to-affective transformation. Cognitive fatigue and psychological exhaustion serve as critical mechanisms linking external stressors to affective responses (Beaudry and Pinsonneault, 2010).

AI anxiety is positively correlated with AI discontinuation intentions, thus H9 was suggested. Affective factors act as key antecedents driving users' technology withdrawal behaviors. This is consistent with the conclusion of Budhathoki et al. (2024), which states that AI anxiety may reduce or even stop the use behavior of AI technology. When users experience anxiety during AI interactions, they are more likely to develop avoidance behaviors and exhibit user resistance (Sapru, 2025).

Study results provide empirical support for H10, confirming that AI dependence serves as a moderator in the relationship between AI anxiety and discontinuation intention. Under conditions of high AI dependence, user behavior becomes increasingly governed by usage inertia and path dependence. Empirical evidence indicates that sustained repetitive engagement reinforces stable behavioral patterns, thereby attenuating the role of affective and cognitive factors in decision-making (Ouellette and Wood, 1998). As a result, even in the presence of anxiety, strong dependency suppresses the tendency toward discontinuation. According to Frenkenberg and Hochman (2025), users with high AI dependence typically exhibit salient usage motivations that attenuate the impact of AI anxiety on discontinuance behavior. These findings contest the straightforward assumption that anxiety uniformly has a link with discontinuation, revealing instead the nuanced and often contradictory mechanisms underlying user decision-making in the context of AI adoption.

### 5.1 Implications and limitations

Against the backdrop of pervasive generative artificial intelligence (AI) deployment, this study aims to advance scholarly understanding of the psychological mechanisms underlying user disengagement behaviors toward generative AI. Through empirical validation of a structured theoretical model, this research delivers substantive theoretical and practical contributions across four key dimensions.

First, this study develops a dual-risk parallel analytical framework, addressing the theoretical inadequacy of single-dimensional perspectives prevailing in extant AI risk scholarship. Prior studies typically examine information authenticity risks and privacy security risks of generative AI in isolation, failing to capture the multi-risk exposure that characterizes users' routine AI interaction experiences. By integrating perceived AI misinformation risk and perceived AI privacy risk into a unified analytical paradigm, the empirical findings identify consistent psychological mechanisms that trigger user cognitive exhaustion and affective strain. This evidence corroborates the multidimensional nature of AIGC risk antecedents, establishes a holistic risk assessment perspective, and lays a solid theoretical foundation for future investigations into risk synergy and interactive effects in AI user behavior research.

Second, this study unpacks the pivotal mediating role of cognitive fatigue within the cognition-affect behavioral chain, refining the psychological transmission mechanism linking risk perception to affective responses. Although the classic cognition-affect-behavior (C-A-B) paradigm delineates the fundamental logical trajectory from cognitive appraisal to behavioral outcomes, it neglects fine-grained intermediate processes governing the cognition-to-affect transformation. The empirical results reveal that AI risk perception positively predicts user anxiety both directly and indirectly via elevated cognitive fatigue. This mediating pathway substantially enhances the explanatory efficacy of the C-A-B framework for high-cognitive-load technological contexts, filling a critical theoretical gap in extant technology behavior literature.

Third, this research validates the moderating effect of AI dependence on the affect-behavior linkage, uncovering the usage inertia constraint mechanism in user technological decision-making. Existing scholarship predominantly emphasizes the proximal predictive power of affective states on user behavioral intentions, while overlooking the boundary conditions imposed by long-term, stable user psychological traits. Consistent with the hypothesized framework, the results indicate that AI dependence attenuates the positive association between user anxiety and disengagement intentions. Specifically, users with high AI dependence tend to follow persistent usage inertia rather than immediate negative affective experiences when making disengagement decisions. This finding extends extant research by shifting the research focus from transient situational emotions to sustained long-term usage patterns. It further provides a compelling theoretical explanation for the technology disengagement paradox—where users retain continuous technology usage despite prominent negative consumption experiences—and clarifies the boundary conditions of technology disengagement behaviors.

Practically, this study yields actionable empirical implications for the responsible design, effective governance, and sustainable development of AIGC technologies. From a user design perspective, AIGC users' negative usage experiences stem from multi-faceted risk perceptions and subsequent cognitive and affective depletion. Accordingly, AIGC product design should transcend singular functional optimization and prioritize the development of secure, trustworthy, and low-cognitive-burden user interfaces and interactive systems. Practitioners can mitigate user cognitive fatigue by embedding trustworthiness indicators and explicit source attribution labels into AIGC outputs, improving information transparency and reducing users' cognitive costs for risk evaluation. From an industrial governance perspective, this study confirms that high AI dependence inhibits users' disengagement behaviors even under adverse usage conditions. AIGC developers and platform operators should therefore establish robust negative feedback response systems to detect and resolve anxiety-triggering usability issues.

The core findings reveal a critical phenomenon: users with high AI dependence persist in usage despite experiencing anxiety, suggesting that platform “stickiness” may reflect lock-in effects rather than genuine user satisfaction. Designers should therefore exercise vigilance against leveraging artificially engineered dependence as a retention strategy, as lock-in-based stickiness risks triggering ethical controversies and incurring long-term reputational damage. To cultivate a healthy and sustainable AIGC ecosystem, practitioners should firmly refrain from deploying dark patterns to manipulate users; instead, they should institute unobstructed exit mechanisms and integrate cognitive-burden and anxiety-reduction metrics into core product evaluation systems, thereby ensuring that user retention is driven by authentic value creation. Furthermore, platforms may establish lightweight, real-time feedback channels to promptly detect and respond to emergent user anxiety.

Notwithstanding its contributions, this study acknowledges several inherent limitations that warrant future research refinement. First, the empirical analysis relies on cross-sectional survey data, which is susceptible to biases such as common method variance and cannot definitively infer causality between variables. Future research should employ longitudinal or experimental designs to not only validate the causal pathways but also dynamically track the evolution of user risk perception and discontinuance behaviors over time.

Second, this study focuses exclusively on generative AI scenarios, which constrains the generalizability of its findings. Alternative AI systems (e.g., recommendation algorithms and automated decision-making systems) differ substantially in risk structure and user interaction logic. Subsequent research could validate and extend the proposed model across diverse AI application contexts. Third, despite the dual-risk framework adopted herein, contemporary AI systems entail additional latent risks, including algorithmic bias and ethical risks, which are not incorporated in the current model. Future studies can expand the dimensions of AI risk perception to construct a more comprehensive multi-risk cognitive framework. Fourth, this study only examines AI dependence as a boundary condition, leaving other individual-level heterogeneities unexamined. Individual traits—particularly age, AI literacy, and professional background—may moderate the affect-behavior transformation process; yet these are not accounted for in the current model. Relatedly, we acknowledge that the sample consists primarily of young, highly educated users; caution is therefore warranted when generalizing our findings to populations with lower AI literacy or greater professional diversity. Future research should employ quota or stratified sampling and introduce individual-level moderators to elucidate the nuanced mechanisms driving heterogeneous user discontinuation intentions.

## 6 Conclusions

Drawing on the classic cognition-affect-behavior (C-A-B) framework, this study systematically investigates the nomological network linking dual-faceted AIGC risk perception (i.e., misinformation and privacy risk perception), domain-specific cognitive fatigue (misinformation fatigue and privacy fatigue), user AI anxiety, and discontinuance intention, while validating the moderating function of AI dependence. The empirical results reveal that AIGC risk perception positively predicts user anxiety through both direct and indirect pathways: heightened risk perception exacerbates cognitive fatigue, which in turn intensifies users' affective anxiety. This dual-path mechanism clarifies how cognitive appraisals shape negative affective states within generative AI interaction settings. Aligned with the fundamental tenets of the C-A-B paradigm, these findings advance extant literature by pinpointing cognitive fatigue as a salient intermediate mechanism that bridges cognitive risk evaluation and affective responses. Furthermore, this study substantiates that AI anxiety acts as a pivotal antecedent of user discontinuance intention. Faced with adverse affective arousal, users tend to engage in avoidant behaviors to mitigate persistent psychological strain. Critically, AI dependence functions as a vital boundary condition for the affect-behavior association. Elevated AI dependence cultivates robust usage inertia, which dampens the positive predictive effect of anxiety on discontinuance intention, thereby demonstrating the multifaceted nature of user decision-making in AI consumption contexts. Collectively, this research deepens theoretical comprehension of the cognitive-affective-behavioral chain under AI risk exposure. It also delivers empirical and practical implications for attenuating user AI discontinuance behaviors via targeted strategies to reduce cognitive fatigue, alleviate affective anxiety, and account for the contingent impact of user AI dependence.

## Data Availability

The datasets presented in this study can be found in online repositories. The names of the repository/repositories and accession number(s) can be found in the article/supplementary material.
